# Functional exploration of the IFT-A complex in intraflagellar transport and ciliogenesis

**DOI:** 10.1371/journal.pgen.1006627

**Published:** 2017-02-16

**Authors:** Bing Zhu, Xin Zhu, Limei Wang, Yinwen Liang, Qianqian Feng, Junmin Pan

**Affiliations:** 1 MOE Key Laboratory of Protein Sciences, Tsinghua-Peking Center for Life Sciences, School of Life Sciences, Tsinghua University, Beijing, China; 2 Center for Biomedical Analysis, Tsinghua University, Beijing, China; 3 Laboratory for Marine Biology and Biotechnology, Qingdao National Laboratory for Marine Science and Technology, Qingdao, Shandong Province, China; Washington University School of Medicine, UNITED STATES

## Abstract

Intraflagellar transport (IFT) particles or trains are composed of IFT-A and IFT-B complexes. To assess the working mechanism of the IFT-A complex in IFT and ciliogenesis, we have analyzed *ift43* mutants of *Chlamydomnonas* in conjunction with mutants of the other IFT-A subunits. An *ift43* null mutant or a mutant with a partial deletion of the IFT43 conserved domain has no or short flagella. The mutants accumulate not only IFT-B but also IFT-Ain the short flagella, which is in contrast to an *ift140* null mutant. The IFT43 conserved domain is necessary and sufficient for the function of IFT43. IFT43 directly interacts with IFT121 and loss of IFT43 results in instability of IFT-A. A construct with a partial deletion of the IFT43 conserved domain is sufficient to rescue the instability phenotype of IFT-A, but results in diminishing of IFT-A at the peri-basal body region. We have further provided evidence for the direct interactions within the IFT-A complex and shown that the integrity of IFT-A is important for its stability and cellular localization. Finally, we show that both IFT43 and IFT140 are involved in mobilizing ciliary precursors from the cytoplasmic pool during flagellar regeneration, suggesting a novel role of IFT-A in transporting ciliary components in the cytoplasm to the peri-basal body region.

## Author summary

Eukaryotic flagella and cilia (interchangeable terms) are microtubule-based cellular structures that project from the cell surface. They play pivotal roles in cell motility and signaling. Ciliary defects are associated with a cohort of human diseases and developmental disorders, termed ciliopathies. The assembly, maintenance and signaling of cilia requires intraflagellar transport (IFT), which is the bidirectional movement of large protein complexes driven by motors within the cilium. IFT protein complexes are composed of two sub-complexes termed IFT-A and IFT-B. IFT43 is a component of IFT-A whose function has not yet been determined. In the model organism *Chlamydomonas*, a unicellular green alga, we have identified an *ift43* null mutant, which reveals that *IFT43* is essential for ciliogenesis. Furthermore, a conserved domain in IFT43 is necessary and sufficient for the function of IFT43. IFT43 directly interacts with IFT121, another IFT-A component, and regulates not only the instability of IFT-A but also its enrichment at the peri-basal body region. In combined studies with other IFT-A mutants, we have found the integrity of IFT-A is important for its stability and proper cellular localization. However, *IFT43* functions distinctively compared to other IFT-A proteins. Loss of *IFT43* or partial deletion of the IFT43 conserved domain induces accumulation of both IFT-A and IFT-B components in the short flagella of the mutant cells. Unexpectedly, we have found that *IFT43* and *IFT140* are required for recruitment of ciliary precursors from the cytoplasmic pool to the flagellar base for ciliogenesis. These data suggest that IFT-A also functions in the transport of ciliary components in the cell body, thus expanding our understanding of the function of IFT-A.

## Introduction

Ciliary assembly and maintenance require intraflagellar transport (IFT), the bidirectional transport within cilia of large protein complexes, termed IFT particles or trains[1, 2]. These complexes concentrate at the peri-basal body region and are transported by kinesin-2 into the cilia and up to the ciliary tip (anterograde IFT) [3–5]. They are returned from the ciliary tip to the base via cytoplasmic dynein 1b/2 (retrograde IFT) [6–9].There are thus various points in the process of IFT where it might be regulated: IFT entry and exit of cilia, turnaround at the ciliary tip, anterograde and retrograde transport of IFT[10–13].

IFT trains are composed of IFT-A and IFT-B complexes. IFT-B, which contains 16 subunits, is required for anterograde transport and thus the delivery of ciliary precursors to the ciliary assembly site at the ciliary tip[14, 15]. IFT-A comprises six polypeptides including IFT144/WDR19, IFT140, IFT139/TTC21b/THM1, IFT122, IFT121/WDR35, and IFT43. A model for the organization of IFT-A has been proposed in whichIFT144, IFT140, and IFT122 form a core complex while the remaining components are peripheral, though several interactions within the complex do not have direct evidence [16, 17].

Analysis of various IFT-A mutants in organisms as diverse as the mouse, fly, worm and *Chlamdyomonas* has revealed multiple functions of IFT-A. (A)For example, with respect to the regulation of ciliogenesis and anterograde transport, cells lacking either IFT144, IFT140 or IFT121 lack cilia entirely or have very short cilia[12, 16, 18–23]. The severe phenotypes of several IFT-A mutants imply that IFT-A may also transport ciliary precursors to build the cilium. IFT144 is involved in transport of membrane proteins including Arl13b in mouse [18], and IFT140 regulates transport of TRPV, an ion channel in the fly [21] and likely guanylyl cyclase in the worm [24]. The IFT-A complex is required for transport of Tulp3 and G protein-coupled receptors [17]. (B)With respect to regulation of IFT entry and turnaround at the ciliary tip, IFT144 connects IFT-A and IFT-B complexes to regulate IFT entry and turnaround at the ciliary base and tip, respectively [12]. (C)In the regulation of retrograde transport, studies in the mouse, flies, worms, *Chlamydomonas*, *Tetrahymena*, and *Trypanosomes* show that mutations in or small RNA interference of IFT-A protein-coding genes cause ciliary accumulation of IFT-B proteins, often accompanied by ciliary bulges or swollen ciliary tips[18–21, 23, 25–29]. In contrast, mutations in IFT-A protein-coding genes usually decrease the amount of IFT-A proteins in cilia. For example, IFT140 is decreased in cilia of*ift122* mutants of mouse and fly[21, 28], IFT139 is reduced in the flagella of *Chlamydomonasift144* mutant [26].

As IFT-A is a protein complex, the phenotypes of IFT-A mutants in ciliogenesis may reflect the specific roles of individual IFT-A subunits, the function of the complex as a whole, or the combination of the two. Mutations in all six human IFT-A genes cause a set of related symptoms with skeletal anomalies accompanied by multiple organ defects, implying the integrity of the entire complex is required for its proper function [22, 30–34].

Characterization of IFT-A mutants in various model organisms has facilitated the elucidation of the function of IFT-A. To our knowledge,*IFT43* is the only IFT-A gene whose mutants have not been characterized and thus its function is not well known. To advance our knowledge of IFT-A, we have thus characterized *ift43* mutants in *Chlamydomonas*. In doing so, we have found a unique role of IFT43 in regulating IFT-A transport, which is distinct from the function of other IFT-A components. We have further determined the organization of the IFT-A complex, and learned that the integrity of the IFT-A complex is required for its stability and its localization at the peri-basal body region. Finally, we have unexpectedly discovered that IFT-A not only functions in cilia but also likely functions in the cell body for transporting ciliary precursors, thus, revealing a role for IFT-A outside the cilia.

## Results

### Characterization of *IFT43* and *IFT43* mutant

**Fig 1:**
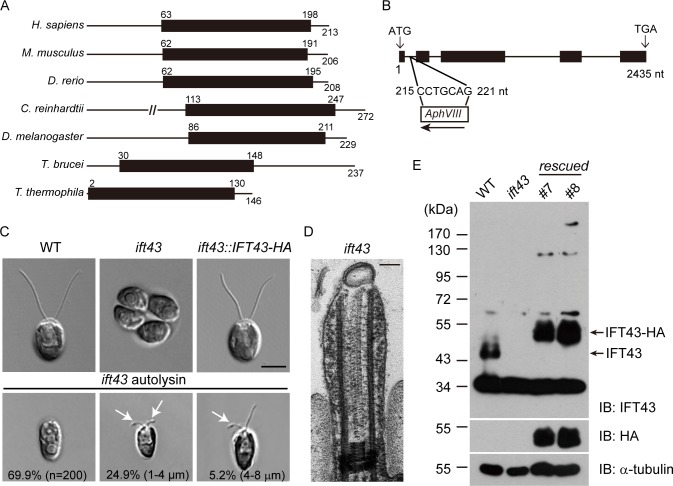
A null mutant of *IFT43* in *Chlamydomonas*. (A) Protein structural domain of IFT43. The numbers indicate the positions of the amino acids. Black bars, IFT43 conserved domain. Protein accession numbers (UniProt): *H*. *sapiens* (Q96FT9); *M*. *musculus* (Q9DA69); *D*. *rerio* (E7F555); *D*. *melanogaster* (Q9VK67); *C*. *reinhardtii* (A8HYP5); *T*. *brucei* (Q586Q5); *T*. *thermophila* (K4ED42). (B) Schematic diagram of the *IFT43* gene (line, intron; black box, exon) showing that the insertion of a foreign DNA fragment (*AphVIII*) in the first intron replacing the nucleotides 215–221 *nt*. The arrow shows the direction of insertion from 5’ to 3’. (C) Wild type (WT), *ift43* and *ift43*::*IFT43*-HA cells were imaged by DIC microscopy. Arrows indicate flagellar bulges. Bar, 5μm. (D)Thin-section EM through the flagellum of *ift43* mutant. Bar, 100nm. (E) Rescue of the *ift43* mutant. HA-tagged *IFT43* gene was transformed into *ift43* cells. Whole cell lysates from *ift43* mutant, two rescued strains, and a WT strain were analyzed by immunoblotting.

IFT43 is regarded as less conserved than other IFT components [16, 35].However, BLAST search revealed that human and several other commonly used model organisms(except for *C*. *elegans*) indeed have IFT43.The IFT43 protein sequence is characterized by the presence of a relatively large conserved amino acid sequence, which we refer to as the IFT43 conserved domain (Fig 1A).To characterize the function of IFT43, we used insertional mutagenesis and obtained an *ift43* mutant. PCR based cloning combined with sequencing showed that nucleotides 215–221 in the first intron of *IFT43* were deleted and replaced by the *AphVIII* DNA fragment (see methods for details)(Fig 1B). The mutant cells formed aggregated colonies, a phenotype often observed with cells lacking flagella or having short flagella (Fig 1C). Indeed, when the cells were released from the mother cell wall by autolysin treatment, most of them were aflagellate while a small percentage of cells formed short flagella with bulges around the flagellar tip. Examination by thin-section EM revealed the accumulation of electron dense materials in the short flagellum that had the characteristic appearance of IFT particles(Fig 1D).

A rabbit anti-IFT43 antibody recognized a protein of ~44 kD in immunoblots of wild type cells but not mutant cells (Fig 1E). HA-tagged *IFT43* was transformed into the *ift43* mutant and the expression of the tagged gene rescued the flagellar phenotype (Fig 1C). Thirty-two percent of the transformants exhibited a rescued phenotype, implying that rescue was a result of incorporation of the transgene, but not disruption of any other genes. Immunostaining of the rescued cells with the anti-HA antibody showed that IFT43-HA was enriched at the peri-basal body region and displayed punctate localization along the length of the flagella(S1A Fig), which is consistent with a previous report on the localization of IFT-A proteins [3].Furthermore, when IFT43-YFP was used in rescue experiments, IFT43-YFP underwent IFT as determined using TIRF microscopy (S1B Fig).

### IFT43 and the integrity of the IFT-A complex regulate the stability of the IFT-A components

**Fig 2:**
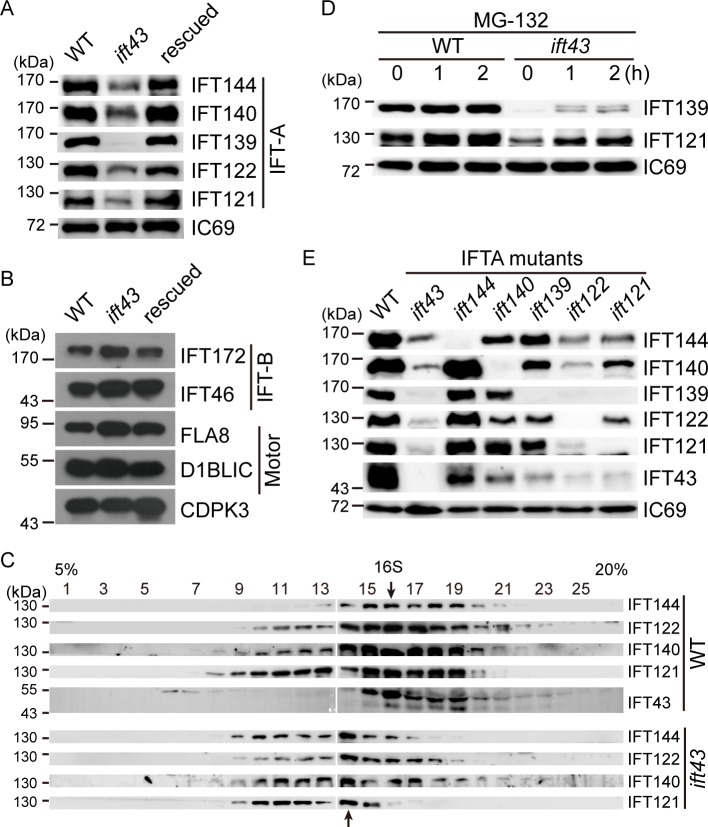
The stability of the IFT-A complex depends on its integrity. (A) Loss of IFT43 decreases the cellular levels of IFT-A proteins. Whole cell lysates were probed by immunoblotting with the antibodies indicated. IC69 was used as a control. (B) Loss of IFT43 does not affect the cellular levels of IFT-B and IFT motors. Whole cell lysates from indicated cells were analyzed by immunoblotting. CDPK3, a calcium dependent kinase 3, was used as a loading control. (C) Loss of IFT43 apparently leads to formation of a smaller IFT-A complex containing IFT144, IFT140, IFT122, and IFT121. Whole cell lysates were subjected to sucrose gradient sedimentation analysis followed by immunoblot analysis. Arrows indicate the peak of the IFT-A complex in WT and *ift43* cells. (D) IFT43 regulates the cellular levels of IFT-A proteins via protein degradation. WT and *ift43* cells were treated with proteasome inhibitor MG-132 for different times followed by immunoblotting. (E) The integrity of IFT-A complex in IFT-A stability. Cell lysates from all IFT-A mutants were probed via immunoblots. Please note that the *ift144* mutant is not a null mutant.

To learn more about the function of IFT43, we investigated its possible role in the formation of the IFT-A complex. First, we examined the cellular levels of IFT proteins by immunoblotting. In comparison to control cells, the levels of the IFT-A proteins IFT144, IFT140, IFT122, and IFT121 in *ift43* mutant cells were greatly decreased while IFT139 was barely detectable (Fig 2A). In contrast, the cellular levels of the IFT-B proteinsIFT172 and IFT46, as well as the FLA8/KIF3B subunit of the anterograde motor kinesin-II and the retrograde motor subunit D1BLICwere relatively unchanged in mutant cells relative to control cells (Fig 2B). These data suggest that IFT43 is essential for the maintenance of the other IFT-A proteins at the correct level in the cell body.

Next, we examined whether reduction of the IFT-A components resulting from the loss of IFT43 would affect the assembly of IFT-A complex. Cell lysates from wild type and *ift43* mutant cells were subjected to sucrose gradient analysis followed by immunoblotting (Fig 2C). In wild type cells, the subunits of IFT-A complex were co-sedimented at fraction 16 as an approximately16 S complex. In mutant cells, IFT144, IFT140, IFT122 and IFT121 appeared to co-migrate as a smaller complex at fraction 14. The formation of a smaller complex would be expected, reflecting the loss of both IFT43 and IFT139. Thus, these data indicate that loss of IFT43 and the concomitant loss of IFT139 do not affect the complex formation by the remaining the IFT-A components, which is consistent with the model that IFT43 and IFT139 are peripheral components of IFT-A [16].

To test whether the decrease of IFT-A proteins in the *ift43* mutant cells was due to protein degradation, wild type and mutant cells were treated with a proteasome inhibitor, MG132, for varying times followed by immunoblotting(Fig 2D). While such a treatment did not affect the amount of IFT139 and IFT121 in wild type cells, it increased the amount of these proteins in mutant cells. These results suggest that IFT-A is protected from protein degradation by the presence of IFT43 or IFT139.

To discern whether IFT43 or IFT139 functions in the stability of the IFT-A components, we generated an *ift139* null mutant (S2 and S3 Figs). *ift139* mutant cells were either aflagellate or had stumpy flagella, indicating that IFT139 is also required for ciliogenesis. Although loss of IFT139 resulted in reduction of the other IFT-A components, the loss of IFT43 by comparison profoundly reduced IFT-A protein levels (Fig 2E). Thus, the decrease in IFT-A proteins observed in the *ift43* mutant is mainly caused by the loss of IFT43. We wondered whether other IFT-A components would affect IFT-A protein levels. To this end, we have also generated mutants defective in the genes encoding other IFT-A components. An *ift144* mutant produced a truncated protein whereas the other mutants were apparently null as revealed by immunoblotting (S2 and S3 Figs).*ift121 and ift122* mutant cells were aflagellate, which is consistent with a previous report [16]. *ift140* mutant cells exhibited short flagella whereas *ift144*mutant cells were aflagellate or had stumpy flagella. Immunoblot analysis showed that the*ift144*mutationonly affected the level of IFT43. In contrast, mutations in *IFT140*, *IFT122* and *IFT121* caused different degrees of reduction of the IFT-A components (Fig 2E). These data suggest that the homeostasis of IFT-A protein levels is maintained by the integrity of the IFT-A complex.

### Organization of the IFT-A complex

**Fig 3:**
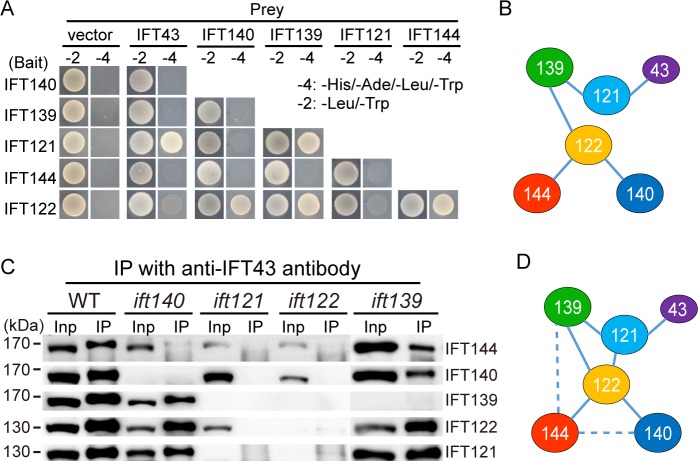
Organization of the IFT-A complex. (A) Analysis of the interactions of IFT-A subunits by yeast two-hybrid assay. The constructs were made by fusing *Chlamydomonas* cDNAs of IFT-A components with the cDNAs of either AD or BD of the GAL4 transcriptional activator. Yeast cells were co-transformed with combinations of IFT-A subunits within the AD and BD vectors, and tested for growth on selective medium. Empty vectors were used as control. (B) An interaction map derived from the yeast two-hybrid assay in (A). (C) Analysis of IFT-A complex formation by antibody pull-down. Cell lysates from WT and various IFT-A mutants as indicated were incubated with anti-IFT43 antibody. The input (Inp) and immunoprecipitates (IP) were resolved by SDS-PAGE followed by immunoblotting with anti-IFT-A antibodies as indicated to the right of each row. (D) An interaction map of the IFT-A complex deduced from antibody pull-down and yeast two-hybrid assays. Dashed line indicates possible interactions. See text for further details and analysis.

As the integrity of the IFT-A complex is required for its stability, and to explain the contribution of each component to the stability of the complex, we set out to determine the architecture of IFT-A. A model for the organization of IFT-A has been proposed previously[16].However, evidence for a direct interaction between proteins within the complex has only been shown for IFT43 with IFT121 and possibly IFT122, and IFT144 with IFT140. By using a yeast two-hybrid assay, we confirmed that IFT43 interacts with IFT121, revealed that IFT139 interacts with both IFT121 and IFT122, and observed that IFT122 interacts with IFT144 and IFT140 (Fig 3A and 3B). Cole and colleagues, however, have only identified an interaction between IFT43 and IFT21 by yeast two-hybrid assay[16]. This difference may be due to differences in the approaches used. Cole and colleagues used a pool library and mating for examination of the interactions while for the data reported here we used pair-wise combinations and co-transformation as the assay.

To verify the above results and find possible additional interactions, we have used anti-IFT43 antibody to pull down IFT-A components from different IFT-A mutant cells (Fig 3C). As expected, in wild type cellsanti-IFT43 antibody pulled down all the components of IFT-A. The results from the pull-down assay using different IFT-A mutant cells support our yeast two-hybrid findings. Bacterial expression of IFT-A proteins has shown that IFT43 also interacts with IFT122[16]. However, in our pull-down in *ift121* mutant cells we did not observe an interaction of IFT43 with IFT122 or the rest of the IFT-A subunits. In the absence of IFT139, the rest of the IFT-A components form a complex, indicating that IFT121 interacts with the core complex formed by IFT144, IFT140 and 122. The question is how IFT121 interacts with the core complex. In the absence of IFT140, IFT121 could still associate with IFT122 and IFT144, indicating IFT121 interacts with either of the two. As IFT122 and IFT121 were similarly pulled down while IFT144 pull-down was greatly reduced, we conclude that IFT121 interacts with IFT122. Previous genetic data suggest that IFT139 interacts with IFT144 [26]. Consistent with this, IFT144 pull-down in the absence of IFT139 was in particular greatly decreased. Thus, based on these data, we have derived a model for the organization of the IFT-A complex(Fig 3D).

The elucidated organization of IFT-A suggests that disruption of the IFT-A complex leads to instability of the IFT-A components. IFT121 interacts with IFT139 and IFT43, which are diminished after loss of IFT121 while the rest of the IFT-A components are only slightly affected. Thus, IFT121 is the organizer of the peripheral components.IFT122 is the core of the IFT-A complex because its loss profoundly reduces all of the rest of the components. Interestingly, though IFT43 is a peripheral component, its loss has a similar effect to the loss of IFT122. This result hints that IFT43 plays a unique role in protecting IFT-A complex from protein degradation.

### IFT43 is distinct from other IFT-A components in regulation of IFT-A transport

**Fig 4:**
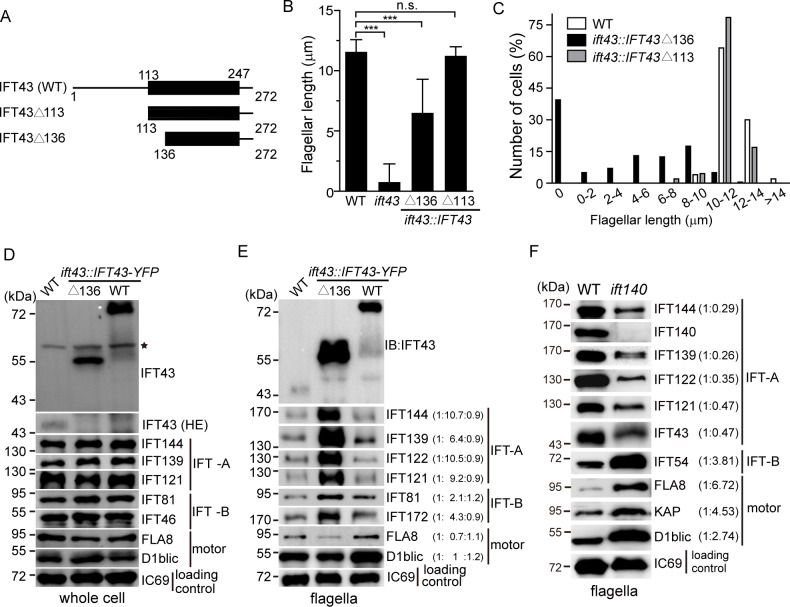
IFT43 plays a distinct role in IFT-A transport. (A) Schematic presentation of the constructs used for rescue of the *ift43* mutant. Numbers indicate the position of the amino acids. Black bars, IFT43 conserved domain. These constructs were tagged with YFP at the C-terminus for the rescue experiments. (B-C) Flagellar length of *IFT43* deletion mutants. *IFT43* gene deletions as indicated were tagged with YFP followed by transformation into *ift43* cells.(B)Average flagellar length of the mutants compared to WT as control. Data are presented as mean ±SD (n = 50 cells).n.s.(*p*>0.05); ***, *p*<0.001(student’s *t*-test).(C)Flagellar length distribution of WT, *IFT43*Δ113 and *IFT43*Δ136 mutant cells. (D) The cellular levels of IFT proteins in the *IFT43*Δ136 mutant are not altered. Immunoblots of cell lysates from *IFT43*Δ136 mutant and control cells were probed with the antibodies as indicated. IFT43 in WT was not detected because of low exposure of the blot in the top panel. A higher exposure (HE) of the blot in the endogenous IFT43 region is shown down below. *, irrelevant bands. (E) Increase of IFT-B as well as well IFT-A in the flagella of the *IFT43*Δ136 mutant. Isolated flagella from *IFT43*Δ136 mutant and control cells were analyzed by immunoblotting. Ratios of protein bands averaged from three independent experiments are in parentheses. (F) Increase of IFT-B and decrease of IFT-A proteins in the flagella of *ift140* mutant cells. Isolated flagella from WT and *ift140* mutant cells were analyzed by immunoblotting.

IFT43 from various species is characterized by a conserved domain (Fig 1A). To determine the function of this domain, we transformed *ift43* null mutant cells with *IFT43* deletion mutants or wild type *IFT43* tagged with YFP. *ift43*Δ113 lacks the N-terminal domain but leaves intact the IFT43 conserved domain while *ift43*Δ136isa partial deletion of the IFT43 conserved domain(Fig 4A). Expression of *ift43*Δ113rescued the flagellar phenotype of *ift43* null mutants (Fig 4B, S4 Fig) and no apparent defect in flagellar regeneration was noted. Thus, the IFT43 conserved domain is sufficient for the function of IFT43 in ciliogenesis. In contrast, *ift43*Δ136 could only partially rescue the flagellar phenotype of the null mutant *ift43*. These mutant cells had either no flagella or short flagella with flagellar bulges (Fig 4B and 4C, S4 Fig), suggesting that an intact IFT43 conserved domain is essential for IFT43 function.

The *IFT43*Δ136mutant cells had longer flagella on average relative to the *ift43* null mutant cells. It has been shown that this mutation does not disturb the interaction of IFT43 with IFT121 [16]. We further examined whether the stability of the IFT-A components is affected in this mutant. As shown in Fig 4D, the mutant cells had levels of the IFT-A components similar to that of wild type controls, which is in sharp contrast to *ift43* mutant cells (Fig 2E).These data highlight the importance of the interaction of IFT43 with IFT121in regulating the stability of the IFT-A components.

Next, we examined the levels of IFT-A proteins in the flagella of *IFT43*Δ136 mutant cells. Flagella isolated from the mutant and control cells were subjected to immunoblot analysis (Fig 4E). IFT-B increased around 3-fold, which is expected as disruption of IFT-A affects retrograde IFT leading to accumulation of IFT-B proteins[36]. It has been reported that IFT-A mutations result in reduction of IFT-A proteins in cilia or flagella[21, 26, 28]. Surprisingly, we found that IFT-A increased as much as 9-fold in average in *IFT43*Δ136 mutant cells(Fig 4E). In the *ift43* null mutant, IFT-A proteins were also accumulated in the flagella (S5 Fig). To pinpoint whether IFT43 plays a unique role in IFT-A regulation, we subsequently analyzed IFT protein levels by immunoblotting of flagella isolated from *ift140* mutant cells (Fig 4F). As expected, IFT-B was increased in the flagella of *ift140* mutant cells. In addition, we observed that IFT motor subunits FLA8, KAP and D1BLIC also increased, which may reflect a possible role for IFT140 in IFT turnaround at the flagellar tip. By contrast, the levels of IFT-A in *ift140* mutant flagella did not increase but rather decreased. Thus, IFT43 plays a unique role among IFT-A subunits in the regulation of IFT-A mediated transport.

### The integrity of the IFT-A complex determines its cellular distribution in the cell body

**Fig 5:**
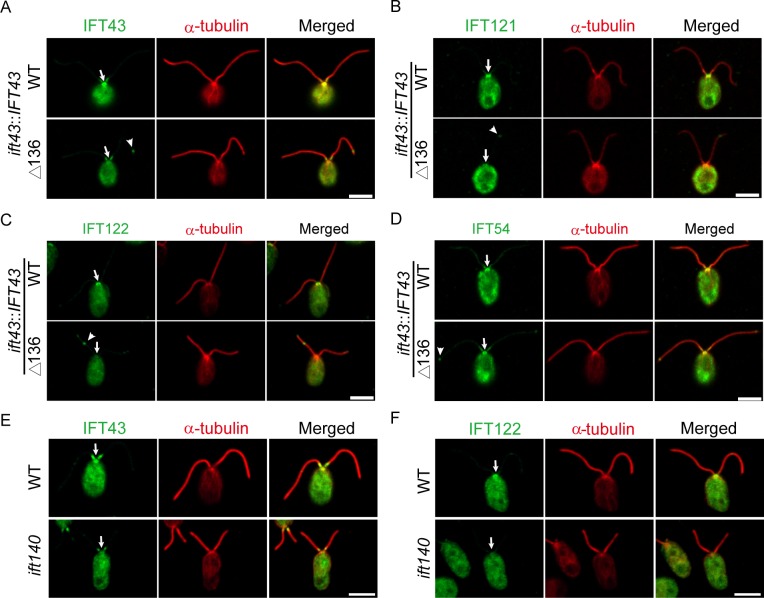
The integrity of the IFT-A complex regulates enrichment of IFT-A at the peri-basal body region. (A-D) The peri-basal body localization of IFT-A but not IFT-B is disrupted in *IFT43*Δ136 mutant. *ift43* mutant cells expressing YFP-tagged IFT43 or *IFT43*Δ136 were immunostained with anti-α-tubulin antibody together with anti-IFT43 (A),anti-IFT121 (B),anti-IFT122 (C), or anti-IFT54antibodies (D). Arrows indicate the flagellar base and arrow heads indicate the flagellar tip. (E-F) The peri-basal body localization of IFT-A proteins is not detected in the*ift140* mutant. WT and *ift140* mutant cells were immunostained with anti-α-tubulin antibody together with anti-IFT43 (E) or anti-IFT122 (F) antibodies. Arrows indicate the flagellar base.

IFT-A and IFT-B proteins are enriched at the peri-basal body region and from there are transported into cilia [3, 37]. As *ift43* mutations alter IFT transport, we next investigated whether they would affect cellular distribution of IFT proteins in the cell body. Unlike the *ift43* null mutation, the *ift43*Δ136 mutation does not affect the cellular level of IFT proteins (Fig 4D), which makes this analysis possible. As expected, IFT-A proteins IFT43, IFT121 and IFT122, and IFT-B protein IFT54 were enriched at the peri-basal body regions in the wild type cells (Fig 5A, 5B, 5C and 5D). In contrast, these IFT-A proteins were undetected or diminished at the peri-basal body region in the *ift43*Δ136 mutant cells while the localization of IFT54 was not altered. Thus, IFT43 is required for the localization of IFT-A in the cell body.

We also asked whether this property is restricted to IFT43 or the integrity of the IFT-A complex. We chose the *ift140* mutant to test this hypothesis as IFT140 does not directly interact with IFT43 and significant levels of IFT-A proteins are still present after loss of IFT140 (Figs 2E and 3D).Similar to *ift43*Δ136 mutant cells, the immunostaining signals of IFT43 and IFT122in *ift140* mutant cells were both undetectable at the flagellar base (Fig 5E and 5F). These data suggest that the integrity of the IFT-A complex is required for proper cellular localization of IFT-A.

### IFT-A is involved in mobilizing flagellar precursors in the cell body for ciliogenesis

**Fig 6:**
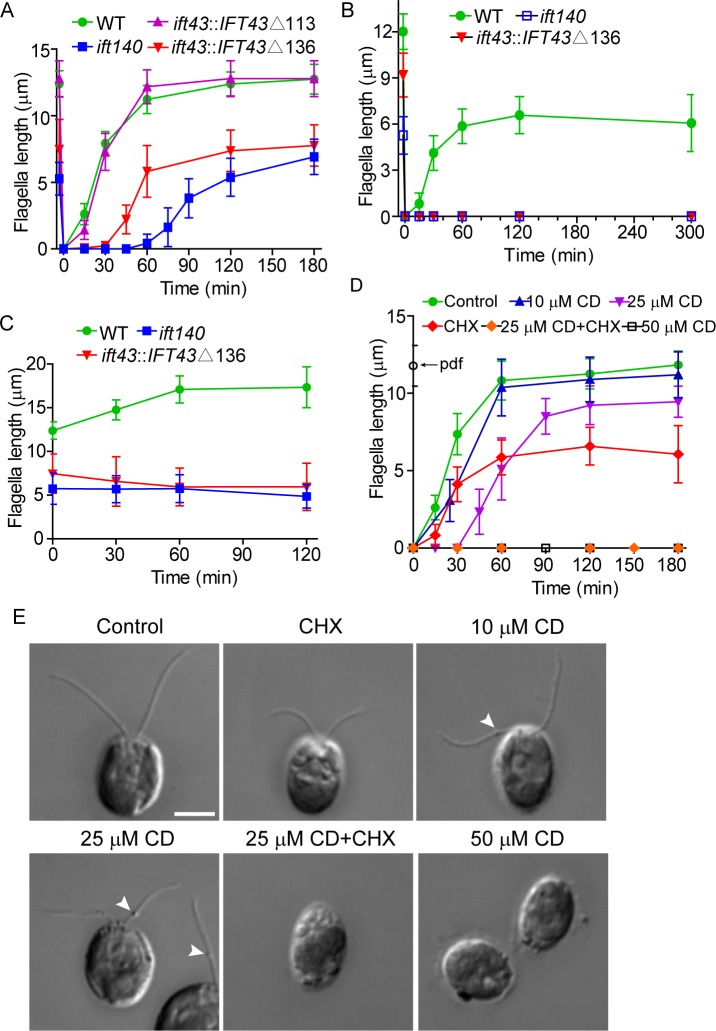
IFT-A is required for mobilizing flagellar precursors in the cell body. (A) Flagellar regeneration in *IFT43*Δ136 and *ift140* mutants exhibits a delay. Flagella were amputated by pH shock to allow flagellar regeneration. Flagellar length data are presented as mean ±SD (n = 50) in this and the following experiments. (B) Flagellar regeneration in *IFT43*Δ136 and *ift140* mutants is blocked in the presence of the protein synthesis inhibitor cycloheximide (10 μg/ml). (C) Flagellar elongation is not induced in *IFT43*Δ136 and *ift140* mutants by LiCl (25 mM) treatment. (D) Effects of ciliobrevin D(CD) on flagellar regeneration of wild type cells. Flagellar regeneration was allowed to proceed in the presence of different concentrations of CD, 10 μg/ml cycloheximide or a combination of the two. Please note, flagellar regeneration at 25 μM CD was blocked in the presence of cycloheximide. pdf, pre-deflagellation flagellar length. (E) Representative images of cells from results shown in (D) at 120 min after deflagellation. Arrow heads indicate flagellar bulges.

The significance of diminished IFT-A enrichment at the basal body is unclear. It may affect flagellar assembly kinetics because the mutant cells may not be able to readily utilize large quantities of IFT-A from the flagellar base during flagellar assembly. To test this, *ift43*Δ136 and *ift140* mutants were deflagellated to allow flagellar regeneration. Consistent with a previous report [38], wild type cells and the mutant *ift43*Δ113 with an intact IFT43 conserved domain rapidly regenerated full length flagella (Fig 6A). The *ift43*Δ136 and *ift140*mutant cells could regenerate flagella to their original length but at a slower rate especially in the*ift140* mutant. Thus, the availability of the proper amount of IFT-A at the flagellar base may play a role in determining the rate of flagellar assembly.

Interestingly, we noticed a delay in the initiation of ciliogenesis in both mutants. Previously, it has been shown that rapid flagellar regeneration after flagellar loss requires precursors from the cytoplasmic pool while new protein synthesis that is triggered by flagellar loss is only required at later stage[38–40]. We reckoned that the delay in flagellar assembly in the mutants is caused by the inability of the cells to mobilize flagellar precursors from the cytoplasmic pool and the delayed flagellar assembly is only triggered when new protein synthesis is available. To test this hypothesis, we treated cells with the protein synthesis inhibitor cycloheximide during flagellar regeneration. Indeed, wild type cells rapidly formed half-length flagella (Fig 6B)as reported [38]. In contrast, flagellar regeneration in *ift43*Δ136 and *ift140* mutants under the same condition was completely blocked. To further test this hypothesis, we examined flagellar elongation induced by LiCl where recruitment of preexisting flagellar precursors but not new protein synthesis is required [41]. As expected, wild type cells elongated flagella normally while flagellar elongation in both mutants did not occur (Fig 6C). Thus, these data suggest that recruitment of flagellar precursors from the cytoplasmic pool is compromised in these mutants.

If IFT-A is involved in the transport of flagellar precursors in the cell body, cytoplasmic dynein is expected to be required. Ciliobrevin D (CD), an inhibitor of dynein, has been shown to inhibit IFT[42, 43]. We subsequently examined the effects of CD on flagellar regeneration in wild type cells (Fig 6D and 6E). At 50 μM CD, ciliogenesis was completely blocked. At 10 μM CD, cells regenerated flagella almost normally as the control cells. However, flagellar bulges could be seen in most of the cells, which reflects IFT inhibition by CD treatment. At 25 μM CD, a delay of ciliogenesis was observed. As expected, ciliogenesis was completely blocked in the presence of cycloheximide at 25 μM CD. Thus, these data lead us to conclude that IFT-A and cytoplasmic dynein function in transport of flagellar precursors in the cell body to the flagellar base.

## Discussion

Human patients with mutations in *IFT43* develop ciliopathies, demonstrating the importance of IFT43 in ciliogenesis or cilia-related function [34]. In this work, we have revealed the working mechanism of IFT43 in regulating ciliary assembly and IFT. By using genetic and biochemical approaches, we have provided direct evidence for the organization of the protein components of IFT-A. Furthermore, we have shown that the integrity of IFT-A is important for the stability of this complex and its proper cellular localization. Most importantly, our data suggest that IFT-A is required for the transport of ciliary precursors in the cell body, revealing new roles of IFT-A outside cilia in ciliogenesis. As IFT was first discovered in *Chlamydomonas*, and IFT is conserved in almost all ciliated organisms, our findings reported here are applicable to IFT studies in other organisms, including mammals.

### IFT43 plays a distinct role in the regulation of IFT-A mediated transport

The generally accepted model of IFT posits that the anterograde motor kinesin-2 moves IFT-B which is associated with IFT-A to the ciliary tip. After remodeling of IFT complexes and motors at the ciliary tip, the retrograde motor cytoplasmic dynein 2/1b carries IFT-A which is associated with IFT-B, and moves back to the ciliary base[15]. Thus, it is expected that disruption of IFT-A would cause ciliary accumulation of IFT-B [36]. Consistent with this, *ift43* mutant cells that still possess short flagella accumulate IFT-B with formation of flagellar bulges. Surprisingly, we found that IFT-A proteins are also increased in the flagella, which is in contrast to previous findings where disruption of IFT-A encoding genes reduces other IFT-A proteins in the cilia. IFT139 is reduced in *Chlamydomonasift144* mutant flagella [26] and IFT140 is undetectable in cilia of fly or mouse in the absence of IFT122 [21, 28]. To discern a unique role of IFT43 in IFT-A transport, we also analyzed an*ift140* mutant. Unlike the *ift43* mutant but consistent with previous findings, the *ift140* mutant shows a decrease of IFT-A proteins in the flagella. Thus, IFT43 functions distinctively in regulating IFT-A transport.

Two mechanisms may explain the flagellar increase of IFT-A resulting from *IFT43* mutations. *IFT43* mutations may disturb tight control of IFT-A entry into cilia to allow increased entry of IFT-A or disrupt the association of IFT-A with cytoplasmic dynein 2/1b leading to IFT-A accumulation. Disruption of the association of IFT-A with cytoplasmic dynein2/1bwould induce an increase of flagellar IFT-A and IFT-Bin similar amounts or more IFT-B than IFT-A. However, we noted that IFT-A increases around 9-foldonaverage while IFT-B increases only around 3-fold. Thus, IFT43 likely controls flagellar entry of IFT-A.

### The effect of the integrity of the IFT-A complex on its stability and cellular localization

Mutations in the six genes encoding the IFT-A polypeptides cause similar ciliopathies resulting in skeletal defects[22, 30–34]. This suggests that the integrity of IFT-A is important for its function.

Our data suggest that the integrity of the IFT-A complex is required for the stability of the IFT-A components. As revealed by our data on the organization of the IFT-A complex, IFT122 serves as a central component of the complex. Its loss results in the disruption of the entire complex followed by protein degradation via the proteasome. Interestingly, loss of the peripheral components results in different degrees of instability of the IFT-A components. The mutant generated by partial deletion of the IFT43 conserved domain is still able to interact with IFT121and thus could rescue the protein instability phenotype that results from a complete loss of IFT43. These data demonstrate that the integrity of the complex is important for IFT-A stability. Interestingly, IFT43 is a peripheral component and its loss causes similar effects to a loss of IFT122. Thus, IFT43 may play a unique role in regulation of IFT-A stability. Because our data show proteasome involvement in the loss of IFT-A components in the *ift43* null mutants, it is likely that binding of IFT43 to the IFT-A complex prevents protein ubiquitination and thus degradation via the proteasome.

In addition, we showed that the integrity of IFT-A is also important for its cellular localization. Mutations in *IFT43* or *IFT140* diminished IFT-A enrichment at the basal body. In *Xenopus*, Jbts17 and CPLANE proteins mediate the recruitment of IFT-A peripheral components (IFT139, IFT121 and IFT43) but not the “core” complex to the ciliary base [44].Because both IFT43 and IFT122, the latter a component of the core complex, are not enriched at the flagellar base in the IFT-A mutants, additional mechanism may be involved. IFT proteins dock onto the basal body transition fibers, a process which may play a role in IFT-A recruitment[45, 46].The IFT-B protein IFT74 is required for relocating IFT-A to the transition fibers from the peri-basal body region, however, it does not affect IFT-A enrichment [37]. In *C*. *elegans* and mammalian cells, transition fiber protein FBF1 regulates IFT-A ciliary entry, but not its localization at the ciliary base [46]. Thus, it is unclear how IFT-A localizes to the basal body region. This is an exciting problem to be addressed by future research.

### Role of IFT-A in ciliogenesis and transport of ciliary precursors in the cell body

IFT-A has been proposed to play a major role in retrograde IFT [36],and recent evidence has revealed that IFT-A is also involved in anterograde transport of ciliary proteins and is required for ciliogenesis. For example, a null mutation of *IFT144* or less strong combined mutations in *IFT144* and *IFT122* block normal ciliogenesis, and IFT144 is required for transport of membrane proteins such as Arl13b into cilia [18].

Because IFT moves ciliary precursors from the ciliary base to the tip for incorporation during cilia formation and maintenance, disruption of IFT within the cilium affects ciliogenesis. However, to be transported into cilia by IFT, ciliary precursors in the cell body need to be delivered to the ciliary base. The mechanism of this delivery is unknown. Theoretically, either a transport mechanism or diffusion would be required. Ciliary proteins are derived from two sources: a preexisting cytoplasmic pool as well as new protein synthesis [38, 39]. In *Chlamydomonas and Tetrahymena*, it has been shown that after ciliary amputation, cells undergoes partial ciliation in the absence of protein synthesis by utilizing precursors from the cytoplasmic pool while complete ciliation requires new protein synthesis[38, 39, 47]. In *Chlamydomonas*, new protein synthesis starts shortly after ciliary amputation and reaches a maximum around 45 min [40]. We have shown that both *ift43* and *ift140* mutants exhibit a delay in flagellar regeneration, which is blocked in the absence of protein synthesis. Similarly, flagellar elongation induced by LiCl where flagellar precursors from the cytoplasmic pool are utilized is also blocked in these two mutants. These data suggest that IFT-A functions in part to transport flagellar precursors from the cytoplasmic pool to the flagellar base. Ciliary membrane proteins are transported from the trans-Golgi network to the ciliary base[48–50]. Axonemal proteins have been found to be associated with vesicles in the cell body that are expected to be delivered to the ciliary base for ciliary entry[51]. The need for delivery of ciliary precursors to the ciliary base is consistent with a possible additional, cytoplasmic transport role for IFT-A in the cell body. However, other possibilities may exist. IFT-A may also function in the cell body to bind and anchor ciliary precursors that are transported to the ciliary base by some other mechanisms. As *ift43* and *ift140* mutants can eventually regenerate short flagella in the presence of protein synthesis, this implies that newly synthesized proteins may move to the ciliary base by diffusion when transport by IFT-A is abrogated in the mutants.

Because cytoplasmic dynein is a microtubule minus end directed motor and the minus ends of the cytoplasmic microtubules face the basal body region, cytoplasmic dynein is expected to be the motor involved in IFT-A transport in the cell body. Consistent with this, we have shown that inhibition of cytoplasmic dynein generates a similar phenotype as the IFT-A mutants that we have analyzed. In *Chlamydomonas*, only one cytoplasmic dynein has been found, that is cytoplasmic dynein 2/1b or IFT dynein [52], which suggests that IFT dynein transports IFT-A in the cell body. This conclusion is supported by a recent report that showed IFT dynein is required for transport of membrane proteins to the peri-basal body region in *Chlamydomonas*[43]. In mammalian cells, there are two types of cytoplasmic dynein: cytoplasmic dynein 1 and IFT dynein[52].Interestingly, tandem affinity purification of IFT-A in mouse has identified cytoplasmic dynein 1 but not IFT dynein[17, 44], raising the possibility that cytoplasmic dynein 1 mediates IFT-A transport in the cell body of mammalian cells.

## Materials and methods

### Strains, culture conditions and special reagents

*Chlamydomonas reinhardtii* wild type strain*21gr* (mt+) (CC-1690) is available from the *Chlamydomonas* Resource Center, University of Minnesota. All mutant strains and transformants were generated from this wild type strain. Cells were grown at 23˚C in liquid R medium for 4–5 days and then transferred to liquid M medium for 2–3 days in Erlenmeyer flasks. The cells were aerated with normal air under a 14:10 hour light-dark cycle. For transformation experiments, the cells were grown in liquid TAP medium for 4 days in constant light. LiCl and cycloheximide were from Sigma (Shanghai, China), MG132 from Selleck (Beijing, China), and Ciliobrevin D from Millipore (USA).

### Insertional mutagenesis, gene cloning and transformation

Previously published protocols were used to generate insertional mutants by transformation of *Chlamydomonas* wild type cells with a ~ 2 kb fragment containing the paromomycin resistance gene *AphVIII*[53]. The disrupted genes were identified by cloning the flanking genomic sequences using RESDA PCR followed by sequencing [54, 55]. The DNA fragment with about 250 nt being deleted from 5’ end has replaced the nucleotides 215–221 of the *IFT43* gene. The insertion information of other IFT-A mutants is provided in the supplemental data. The *IFT43* gene with an approximately 1.5 kb fragment upstream of the start codon was cloned by PCR and verified by sequencing. To rescue the mutant phenotype of *ift43*, the *IFT43* gene was tagged with 3×HA or YFP followed by a RUBISCO terminator. Deletion mutants of the *IFT43* gene were constructed based on the wild type *IFT43* gene construct. These constructs were cloned into a modified vector pHyg3 harboring a hygromycine B resistance gene[56]. The final constructs were linearized with ScaI and transformed into the *ift43* mutant using electroporation[57]. The cDNAs for the IFT-A genes were cloned from a *Chlamydomonas* cDNA library (Takara, Dalian, China).

### Flagellar isolation and flagellar phenotype analysis

Flagellar isolation or flagellar regeneration after deflagellation by pH shock were as previously described [58]. Purified flagella were resuspended in buffer A (50 mM Tris (pH 7.5), 10 mM MgCl2, 1 mM EDTA, 1 mM DTT) containing a protease inhibitor cocktail (complete-mini EDTA free, Roche), 0.1% NP40 and 20 μM MG132. To induce flagellar elongation, LiCl was used at a final concentration of 25 mM. To block protein synthesis during flagellar regeneration, 10 μg/ml cycloheximide was added at 20 min before deflagellation. For flagellar phenotype analysis, cells were fixed with 1% glutaraldehyde and imaged by a Zeiss DIC microscope (Zeiss Axio) equipped with a CCD camera (QuantEM 512SC, Photometrics, USA)using a 100x objective. Images were exported and processed using Photoshop and Illustrator (Adobe). For palmelloid cells, the cells were treated with gametic autolysin to release the daughter cells followed by DIC microscopy. A previous procedure was used to examine the ultrastructure of the flagella by thin section EM [55, 59].

### Yeast two-hybrid assay

cDNAs of IFT-A genes from *Chlamydomonas* were cloned into yeast expression vectors pGADT7 and pGBKT7, respectively. All possible pair combinations of the IFT-A gene constructs were co-transformed into yeast strain AH109. After transformation, the cells were grown at 30°C for 2–3 days on selection medium SD lacking leucine, tryptophan, histidine, and adenine (SD, -Leu, -Trp, -His, -Ade) or lacking leucine and tryptophan (SD, -Leu, -Trp).

### Antibodies

The information for the primary antibodies used for immunoblotting and immunostaining can be found in S1 Table. The antibodies for IFT54, and all IFT-A proteins were made during this study and the information can be found in S2 Table. For the secondary antibodies used in immunostaining, Texas Red-conjugated goat anti-mouse IgG (1:200), Alexa-Fluor-488-conjugated goat anti-rabbit IgG (1:200),Alexa-Fluor-488-conjugated goat anti-rat IgG (1:200) (Molecular Probes) were used.

### Immunoprecipitation and sucrose gradient analysis

For immunoprecipitation, 1X10^9^ cells were lysed in 1mlbufferA-IP (20mM HEPES (pH 7.2), 5mM MgCl_2_, 1mM DTT, 1mM EDTA (pH 7.5), 150mM NaCl, 20mM β-glycerol phosphate, 0.1mM Na_3_VO_4_, 10mM NaF, and 5% glycerol, 20μM MG132).15 μl anti-IFT43 antibody was used for immunoprecipitation. For sucrose gradient analysis, a previously published protocol was followed [60]. Approximately 25–26 fractions were collected.

### Immunoblotting and immunofluorescence microscopy

Previously published protocols were followed [61]. For immunofluorescence, cells were fixedin4% paraformaldehyde, permeabilized with 0.5% NP40, resuspended in MT buffer, and attached to a 10 well slide previously coated with 0.1% polyethyleneimine and extracted with methanol at -20°C for 10 min. The cells were then incubated with primary antibodies at 37°C for 4hrs or at 4°C overnight followed by washing and incubation with the secondary antibodies at 37°Cfor 4hrs.The samples were viewed on a Zeiss LSM780 META Observer Z1 Confocal Laser Microscope. Images were acquired and processed by ZEN 2009 Light Edition (Zeiss) and Photoshop software (Adobe).

### Live imaging of IFT

Cells were immobilized on coverslips coated with 1% polylysine. Images were acquired at room temperature on a Nikon microscope (A1RSi) equipped with a100×, 1.49 N.A. TIRF objective and a cooled EM charge-coupled device (CCD) camera (ORCA-flash 4.0, Hamamatsu). Images were analyzed with NIS-Elements software. Kymographs and videos were generated using Image J (NIH, USA)and the IFT speed and frequency were accordingly calculated.

## Supporting information

S1 Table Antibodies used in this study. (DOCX)

S2 Table Information on newly made antibodies used in this study. (DOCX)

S1 Fig IFT43 undergoes IFT. (A) IFT43 shows typical localization of IFT proteins with enrichment at the peri-basal body region and a punctate distribution throughout the flagellum. WT and *ift43*::*IFT43*-HA cells were immunostained with anti-HA and anti-α-tubulin antibodies, respectively. Bar, 5μm. (B) Kymographs of IFT43-YFP. TIRF microscopy was used to observe movement of IFT43 in a strain expressing *IFT43*-YFP in the *ift43* mutant background. The image of the recorded flagellum is on the left side of the kymograph. The expression of IFT43-YFP was further confirmed by immunoblotting with antibodies against GFP and α-tubulin. IFT43-YFP moved at 2.13 and 3.48 μm/s for anterograde and retrograde transport, respectively.
(TIF)

S2 Fig Foreign DNA insertion sites of the IFT-A mutants. Schematic presentations of gene structures and insertion sites of foreign DNA fragment. IFT-A mutants including *ift144*, *ift140*, *ift139*, *ift122* and *ift121* were generated by insertional mutagenesis (See Methods). The gene flanking sequences were identified by PCR and sequencing. The numbers indicate positions of the nucleotides in individual genes. Black box, exon; line, intron; nucleotides shaded in yellow, gene flanking sequences; nucleotides shaded in gray, flanking sequences of foreign DNA inserts.
(TIF)

S3 Fig Characterization of IFT-A mutants. (A) IFT-A mutants as indicated were analyzed by immunoblotting of whole cell lysates with wild type (WT) cells as control. The blots were probed with individual IFT-A antibody, respectively. (B-F) DIC images of cells from different mutants. (B) *ift144* mutant. (C) *ift140* mutant. (D) *ift139* mutants. (E) *ift122* mutant. (F) *ift121* mutant. Arrows indicate flagellar bulges. Bar, 5 μm.
(TIF)

S4 Fig Flagellar phenotypes of IFT43 partial deletion mutants. Immunoblot of *ift43* deletion mutants. Wild type *IFT43* gene or its mutant variants tagged with YFP were expressed in *ift43* null mutant. Whole cell lysates from the transgenic strains were probed with anti-GFP and IC69 antibodies with WT and *ift43* null mutant cells as control. DIC images of representative cells from *IFT43*Δ136 mutant and WT strain. Arrows indicate flagellar bulges. Bar, 5 μm. EM section of the flagellar bulge region. Accumulated electron dense materials can be seen between the flagellar membrane and the outer doublet microtubules. Bar, 100 nm.
(TIF)

S5 Fig Accumulation of IFT-A and IFT-B in the flagella of *ift43* null mutant. (A) IFT-B protein IFT172 accumulates in the flagellar bulges of *ift43* mutant. WT and *ift43* cells were immunostained with anti-IFT172 antibody followed by fluorescence and DIC microscopy (left panels). Statistics of the flagellar phenotypes of WT and *ift43* cells is presented in the right panel. All flagellar bulges were stained with IFT172 antibody. 50 cells were analyzed. Bar, 5μm. (B) IFT-A protein IFT144 accumulates in the flagellar bulges of *ift43* mutant. Similar analysis as shown in (A) was performed. The cells were stained with anti-IFT144 antibody. Bar, 5μm.
(TIF)
