# Supplementary material for: Functional exploration of the IFT-A complex in intraflagellar transport and ciliogenesis
Source: PLoS Genet. 2017 Feb 16;13(2):e1006627. doi: 10.1371/journal.pgen.1006627 (PMC5336300; doi:10.1371/journal.pgen.1006627)
Supplement: S1 Table — (DOCX) [file pgen.1006627.s001.docx]

**S1 Table. Antibodies used in this study**

| Antibody | Dilution | | Reference or source |
| --- | --- | --- | --- |
|  | **WB** | **IF** |  |
| Rat anti-HA | 1:1000 | 1:50 | Roche |
| Mouse anti-α-tubulin | 1:2500 | 1:200 | Sigma |
| Rabbit anti-GFP | 1:2000 | NA | Abmart |
| Mouse anti-IC69 | 1:20000 | NA | Sigma |
| Mouse anti-IFT139 | 1:10000 | 1:50 | Cole et al., 1998 |
| Mouse anti-IFT172 | 1:2000 | 1:50 | Pederson et al., 2005 |
| Mouse anti-IFT81 | 1:1000 | 1:50 | Cole et al., 1998 |
| Rabbit anti-D1bLIC | 1:1000 | 1:50 | Meng et al., 2016 |
| Rabbit anti-KAP | 1:1000 | NA | Liang et al., 2014 |
| Rabbit anti-FLA8 | 1:2000 | NA | Liang et al., 2014 |
| Rabbit anti-FLA10 | NA | 1:50 | Cole et al., 1998 |
| Rabbit anti-IFT54 | 1:1000 | 1:50 | This study |
| Rabbit anti-IFT46 | 1:1000 | NA | Dr. Kaiyao Huang |
| Rabbit anti-IFT43 | 1:1000 | 1:50 | This study |
| Rabbit anti-IFT144 | 1:1001 | 1:51 | This study |
| Rabbit anti-IFT140 | 1:1002 | 1:52 | This study |
| Rabbit anti-IFT122 | 1:1003 | 1:53 | This study |
| Rabbit anti-IFT121 | 1:1004 | 1:54 | This study |
| Rabbit anti-CDPK3 | 1:1005 | NA | Liang et al., 2013 |

IF, immunofluorescence; NA, not applicable; WB, Western blot.
