# Supplementary material for: Functional exploration of the IFT-A complex in intraflagellar transport and ciliogenesis
Source: PLoS Genet. 2017 Feb 16;13(2):e1006627. doi: 10.1371/journal.pgen.1006627 (PMC5336300; doi:10.1371/journal.pgen.1006627)
Supplement: S2 Table — (DOCX) [file pgen.1006627.s002.docx]

**S2 Table. Information on newly made antibodies used in this study**

| Protein | Antigen | Affinity Tag | Host/Source |
| --- | --- | --- | --- |
| IFT43 | aa 1-272(full length) | N-terminal His-Tag | Rabbit (YingJi, Shanghai) |
| IFT121 | aa 1002-1224(C-terminal) | N-terminal His-Tag | Rabbit (YingJi, Shanghai) |
| IFT122 | aa 1050-1234(C-terminal) | N-terminal His-Tag | Rabbit (YingJi, Shanghai) |
| IFT140 | aa 1182-1384(C-terminal) | N-terminal MBP tag | Rabbit (B&M, Japan) |
| IFT144 | aa 137-455(N-terminal) | N-terminal His-Tag | Rabbit (YingJi, Shanghai) |
| IFT54 | aa 123-384(C-terminal) | C-terminal His-Tag | Rabbit (B&M, Japan) |
