# Supplementary figures and images for: Functional exploration of the IFT-A complex in intraflagellar transport and ciliogenesis
Source: PLoS Genet. 2017 Feb 16;13(2):e1006627. doi: 10.1371/journal.pgen.1006627 (PMC5336300; doi:10.1371/journal.pgen.1006627)

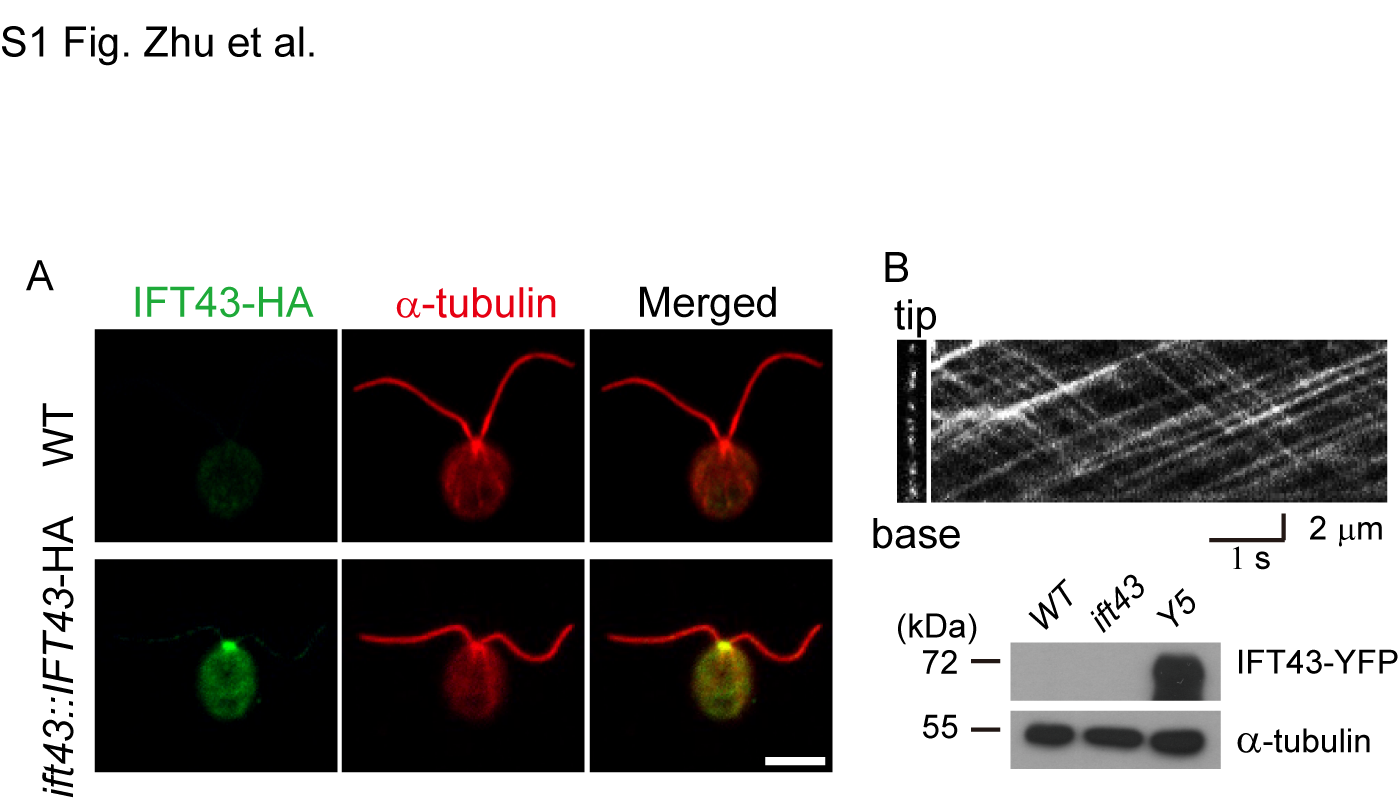

Supplement: S1 Fig — (A) IFT43 shows typical localization of IFT proteins with enrichment at the peri-basal body region and a punctate distribution throughout the flagellum. WT and ift43::IFT43-HA cells were immunostained with anti-HA and anti-α-tubulin antibodies, respectively. Bar, 5μm. (B) Kymographs of IFT43-YFP. TIRF microscopy was used to observe movement of IFT43 in a strain expressing IFT43-YFP in the ift43 mutant background. The image of the recorded flagellum is on the left side of the kymograph. The expression of IFT43-YFP was further confirmed by immunoblotting with antibodies against GFP and α-tubulin. IFT43-YFP moved at 2.13 and 3.48 μm/s for anterograde and retrograde transport, respectively. (TIF) [file pgen.1006627.s003.tif]

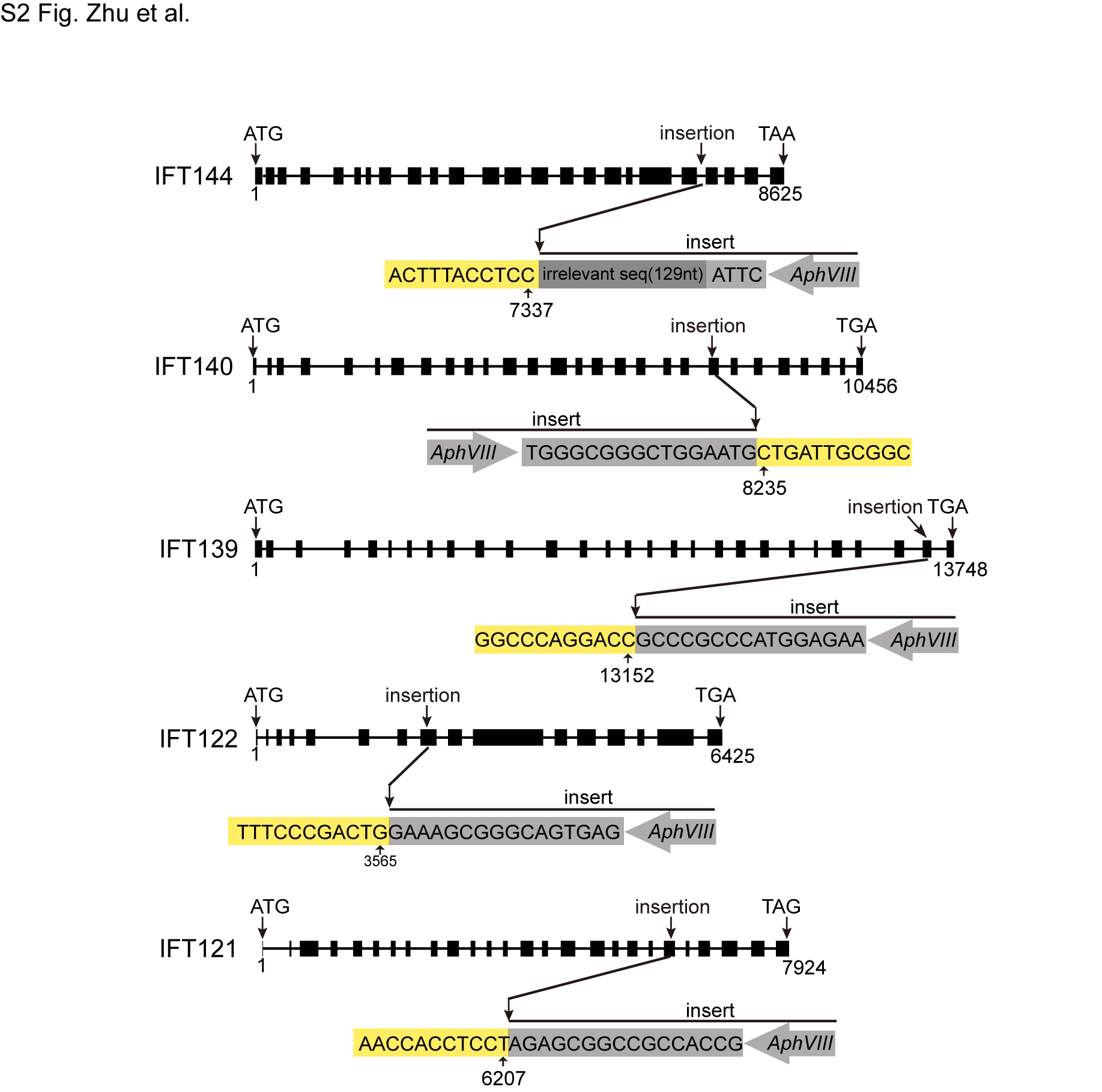

Supplement: S2 Fig — Schematic presentations of gene structures and insertion sites of foreign DNA fragment. IFT-A mutants including ift144, ift140, ift139, ift122 and ift121 were generated by insertional mutagenesis (See Methods). The gene flanking sequences were identified by PCR and sequencing. The numbers indicate positions of the nucleotides in individual genes. Black box, exon; line, intron; nucleotides shaded in yellow, gene flanking sequences; nucleotides shaded in gray, flanking sequences of foreign DNA inserts. (TIF) [file pgen.1006627.s004.tif]

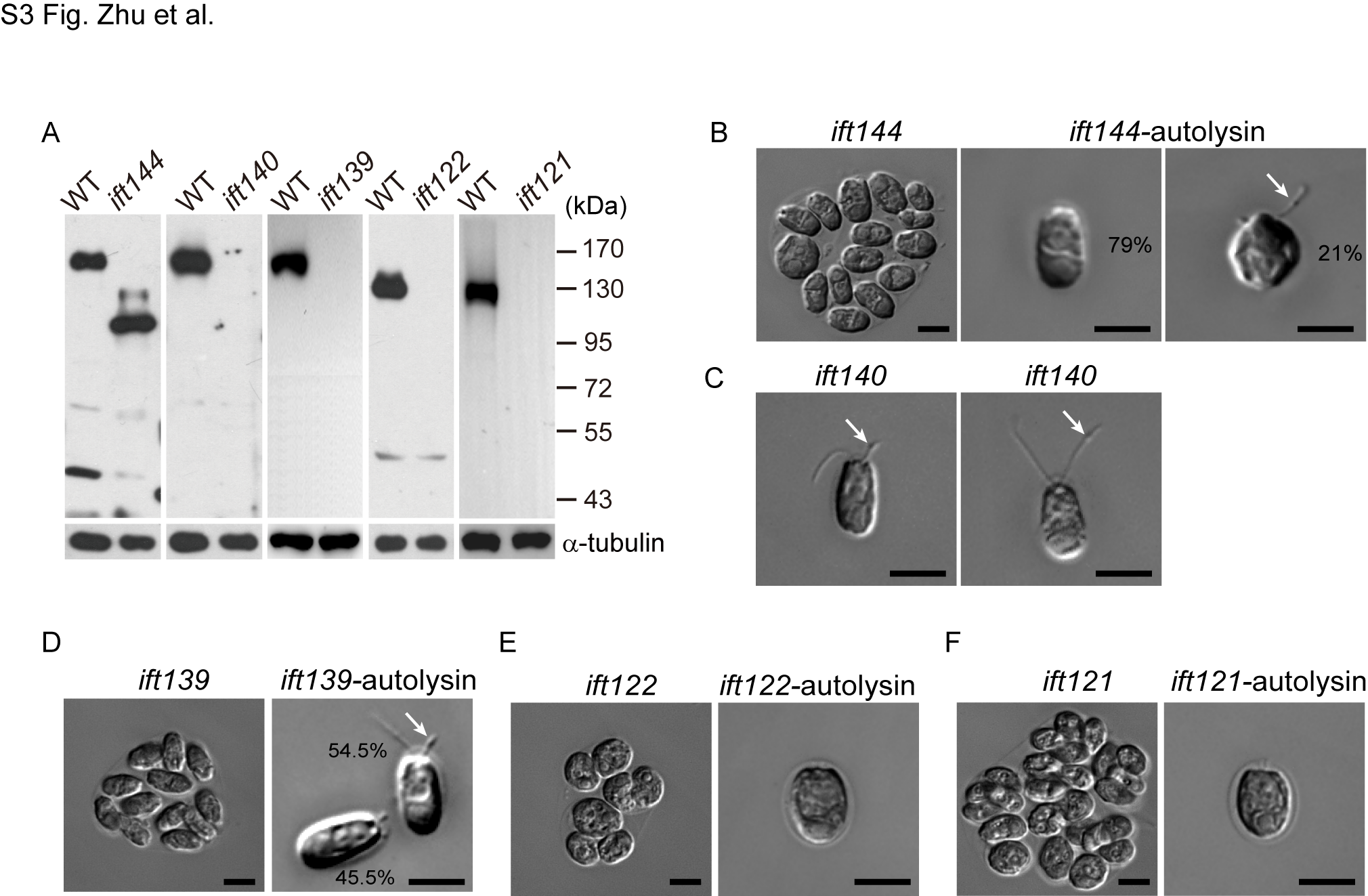

Supplement: S3 Fig — (A) IFT-A mutants as indicated were analyzed by immunoblotting of whole cell lysates with wild type (WT) cells as control. The blots were probed with individual IFT-A antibody, respectively. (B-F) DIC images of cells from different mutants. (B) ift144 mutant. (C) ift140 mutant. (D) ift139 mutants. (E) ift122 mutant. (F) ift121 mutant. Arrows indicate flagellar bulges. Bar, 5 μm. (TIF) [file pgen.1006627.s005.tif]

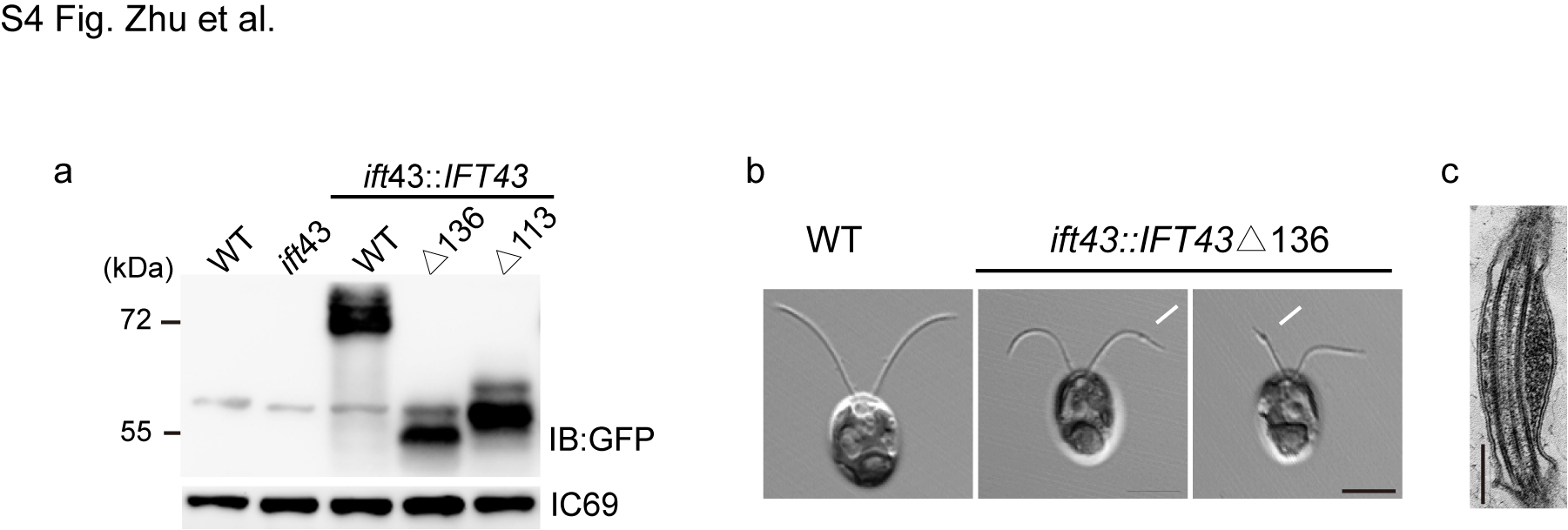

Supplement: S4 Fig — Immunoblot of ift43 deletion mutants. Wild type IFT43 gene or its mutant variants tagged with YFP were expressed in ift43 null mutant. Whole cell lysates from the transgenic strains were probed with anti-GFP and IC69 antibodies with WT and ift43 null mutant cells as control. DIC images of representative cells from IFT43Δ136 mutant and WT strain. Arrows indicate flagellar bulges. Bar, 5 μm. EM section of the flagellar bulge region. Accumulated electron dense materials can be seen between the flagellar membrane and the outer doublet microtubules. Bar, 100 nm. (TIF) [file pgen.1006627.s006.tif]

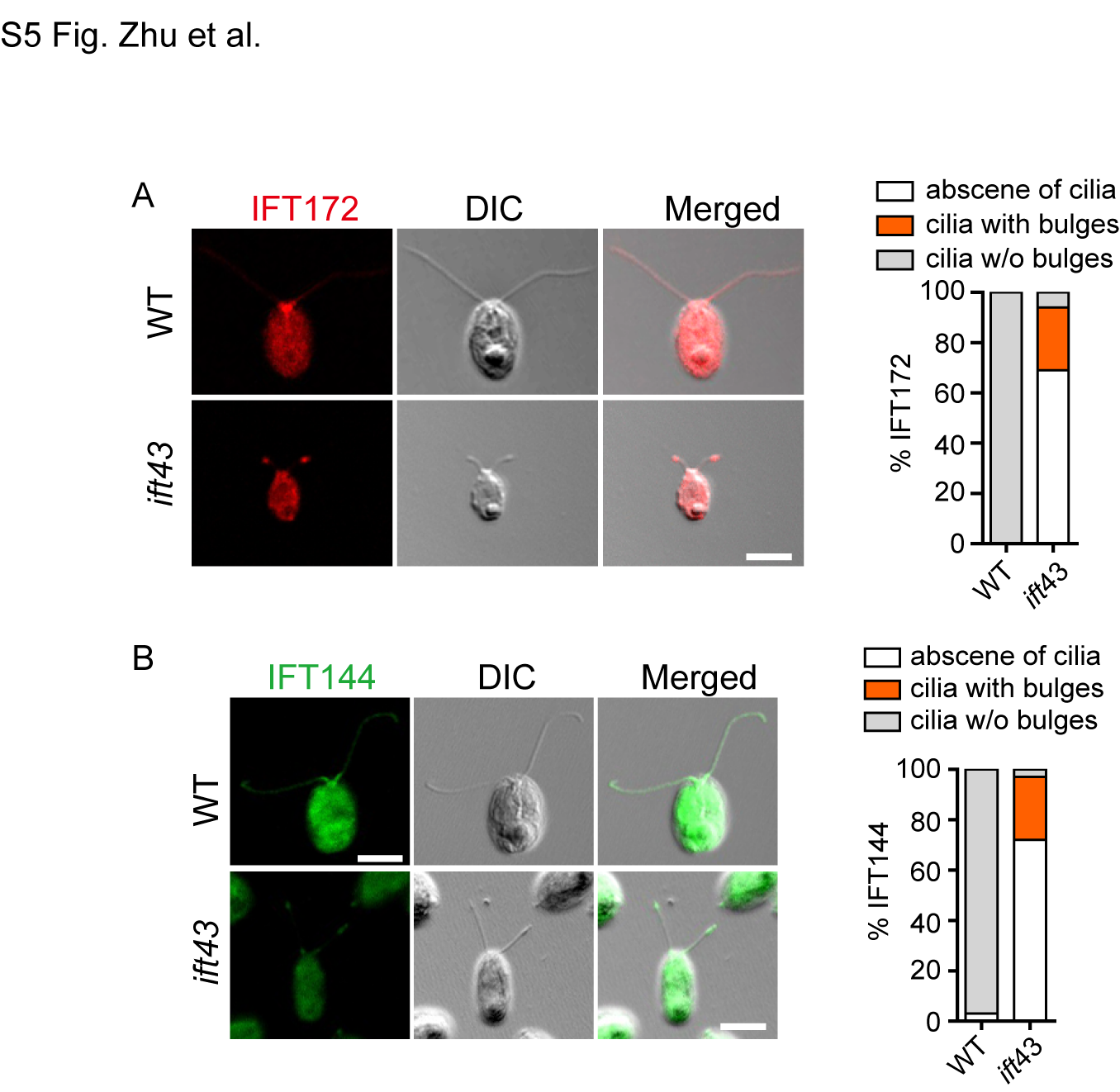

Supplement: S5 Fig — (A) IFT-B protein IFT172 accumulates in the flagellar bulges of ift43 mutant. WT and ift43 cells were immunostained with anti-IFT172 antibody followed by fluorescence and DIC microscopy (left panels). Statistics of the flagellar phenotypes of WT and ift43 cells is presented in the right panel. All flagellar bulges were stained with IFT172 antibody. 50 cells were analyzed. Bar, 5μm. (B) IFT-A protein IFT144 accumulates in the flagellar bulges of ift43 mutant. Similar analysis as shown in (A) was performed. The cells were stained with anti-IFT144 antibody. Bar, 5μm. (TIF) [file pgen.1006627.s007.tif]
